# Primary lymphoma of the uterine horn in a Lhasa Apso dog

**DOI:** 10.1186/2046-0481-66-24

**Published:** 2013-12-10

**Authors:** Ji-Seung Ko, Han-Jun Kim, Su Han, Sun-Hee Do

**Affiliations:** 1 Department of Veterinary Clinical Pathology, College of Veterinary Medicine and Veterinary Science Research Institute, Konkuk University, Seoul 143-701, Republic of Korea; 2 Han Su Animal Clinic, Seoul 122-923, Republic of Korea

**Keywords:** Marginal zone B-cell lymphoma, Canine, Uterus, CD79a

## Abstract

Primary lymphomas of the canine female genital tract are uncommon tumours. A 9-year-old intact female Lhasa Apso dog presenting with a closed pyometra underwent an ovariohysterectomy (OHE), and the hyperplastic uterine horn along with multiple follicular cysts on the right ovary was examined by histological analysis. Severe infiltration of medium-sized lymphocytes with strong positive immunoreactivity for CD79a and numerous anaplastic features was detected in the unilateral uterine horn, and the dog was diagnosed as having extranodal marginal zone B-cell lymphoma (MZBCL). The present case reports an extremely rare occurrence of primary lymphoma involving the uterine horn in a dog and describes histological characteristics of the tumour for definite diagnosis.

## Background

Uterine tumours are rare in dogs and typically include leiomyomas, leiomyosarcomas, adenomas, adenocarcinomas, and fibromas [1]. Although lymphoma, especially non-Hodgkin’s lymphoma, is the most common malignant tumour in dogs [1], lymphomas of the canine genital tract are rarely reported. In animals, lymphoma is classified by anatomical location as multicentric, mediastinal, alimentary, and extranodal lymphoma, and the most common type in dogs is the multicentric type [1]. Usually, tumour cells originate in lymph nodes, and enlarged peripheral lymph nodes are present in the patient; however, extranodal lymphomas affect the skin, bowel, bone, brain, and, rarely, the female genital tract. Biopsy of affected lymph nodes or organs could confirm the diagnosis.

Uterine mucosa is a type of mucosa-associated lymphoid tissue (MALT), which is a specific form of peripheral lymphoid tissue, and extranodal marginal zone B-cell lymphoma (MZBCL) can be derived from MALT within various locations, including the intestines, uterus, salivary glands, lungs, thyroid, and orbita [2]. In humans, chronic inflammatory conditions occasionally lead to primary extranodal MALT-type MZBCL [3].

This patient was diagnosed with extranodal MALT-type MZBCL in the uterus by histopathological and immunohistochemical analysis. This report presents a rare case of primary uterine lymphoma in a dog and describes the diagnostic approach for adequate treatment and prognostic evaluation.

## Case presentation

A 9-year-old intact female Lhasa Apso dog presented to the animal hospital for mild lethargy and anorexia. No significant changes were detected by complete blood count (CBC) and serum chemistry. A hyperplastic uterus was identified on radiologic imaging, and a closed pyometra was suspected; thus, ovariohysterectomy (OHE) was performed. On gross appearance, the right ovary had multiple follicular cysts, and the connected uterine horn showed mild endometrial hyperplasia.

The resected ovary and uterus were fixed in 10% neutral buffered formalin, embedded in paraffin, and processed routinely with haematoxylin and eosin (HE) stain. For immunophenotyping of lymphocytes, immunohistochemical analysis was performed using the Vectastain® Elite ABC-Peroxidase kit^a^ according to the manufacturer’s instructions. Rabbit monoclonal anti-CD3^b^ (1:100) and mouse monoclonal anti-CD79a^c^ (1:200) were used as primary antibodies. The antibody reaction was visualised using diaminobenzidine peroxidase substrate (DAB).^d^ The sections were counterstained with Mayer’s haematoxylin, dehydrated, and mounted.

**Figure 1:**
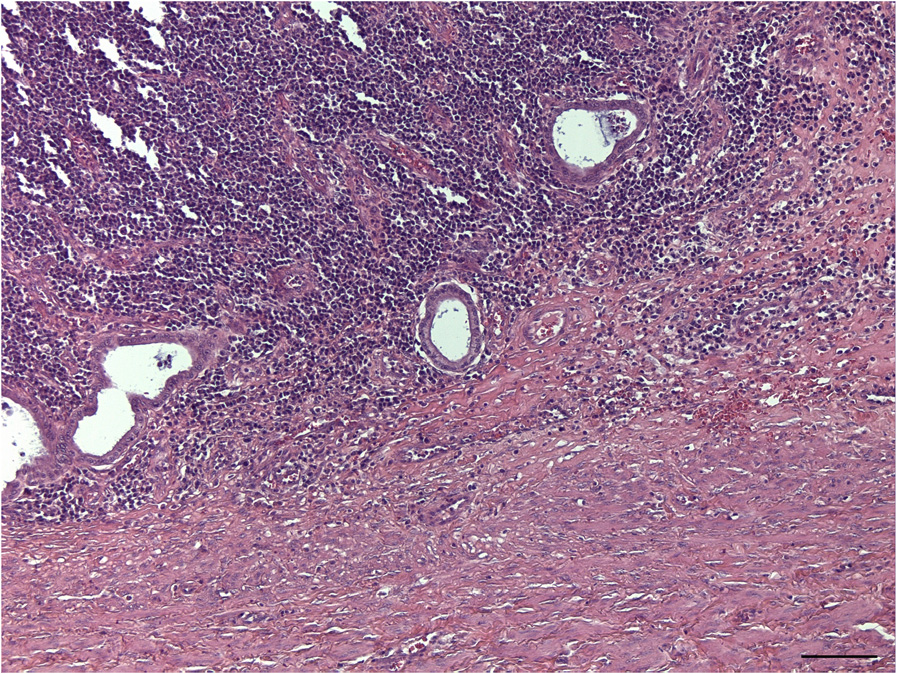
Massive infiltration of lymphocytes in the endometrial lesion accompanying muscular infiltration (haematoxylin and eosin, bar = 100 μm).

**Figure 2:**
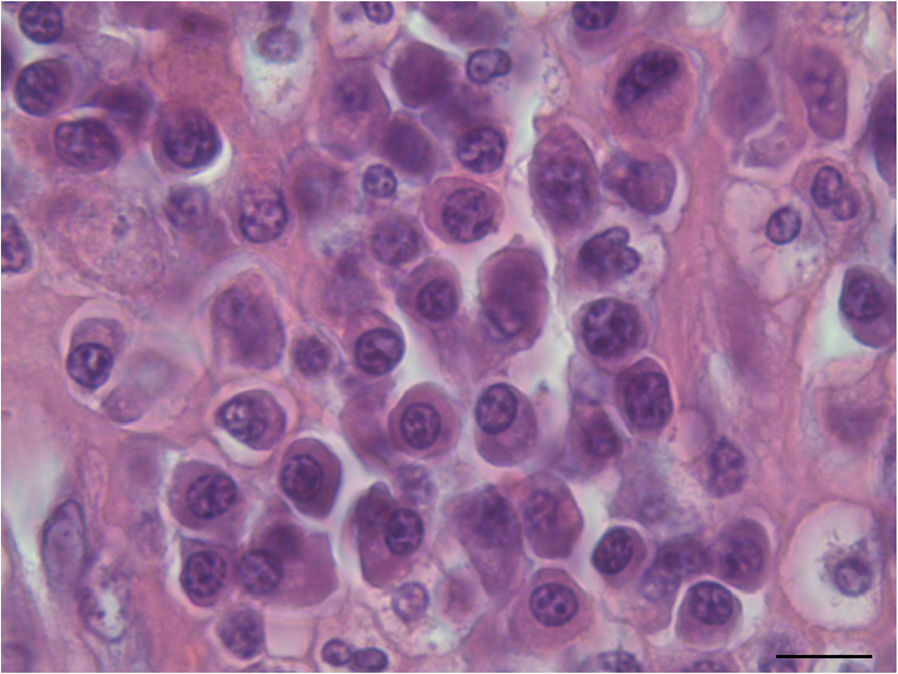
**Morphologic characteristics of tumour cells.** The cells had relatively abundant cytoplasm and pleomorphic central to eccentric nuclei. The chromatin is coarse in general, and multiple prominent nucleoli were observed (haematoxylin and eosin, bar = 10 μm).

**Figure 3:**
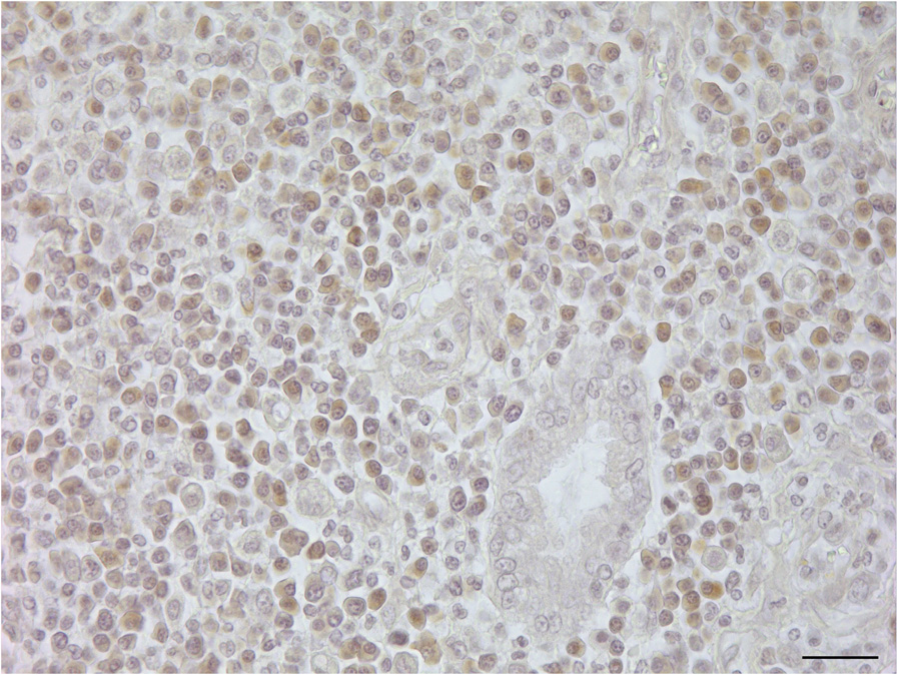
**Positive immunoreactivity for CD79a in the cytoplasm of lymphocytes.** Mayer’s haematoxylin counterstaining (bar = 50 μm).

In HE stain, the uterine horn showed massive infiltration of basophilic cells, and the disappearance of endometrial glands with moderate haemorrhage was noted. The basophilic infiltrates partially invaded the muscular layer (Figure 1). Under higher magnification, the round basophilic cells were identified as medium-sized lymphocytes with a moderate nuclear:cytoplasmic (N:C) ratio and multiple prominent nucleoli (Figure 2). Previously reported descriptions supporting a marginal zone (MZ) B-cell origin include neoplastic cells possessing a relatively abundant amount of cytoplasm, a low N:C ratio, eccentric nuclei, and distinct nucleoli [4]. The neoplastic cells in this case displayed strong immunoreactivity for CD79a (Figure 3) and a negative reaction for CD3 (data not shown), indicating a lymphoma of B-cell origin.

The patient recovered after OHE, and no additional occurrence or systemic metastasis of lymphoma was found.

## Conclusions

Generally, canine lymphomas occur in 5- to 11-year-old (middle-aged) dogs, and 84% of canine lymphomas are a multicentric form by anatomical classification [1]. Primary lymphoma in the female genital tract is extremely rare in both dogs and humans.

In humans, extranodal MZBCL usually arises from gastric mucosa and its MALT [2]. The female genital tract is also rich in mucosa, and MALT has been identified in this location [5]. In humans, primary extranodal MALT-type lymphomas involving the ovary and uterus account for 2% of all extranodal primary lymphomas [2], and two cases have been reported in the fallopian tube [2,6].

Cytologically, the lymphocytes in this case showed cellular features consistent with previously described characteristics of MZBCL, which include a ‘fried egg’ appearance due to an abundant amount of cytoplasm, eccentric nuclei, and occasional visible nucleoli [4]. Subclasses of MZBCL include extranodal MZBCL of MALT, splenic MZBCL, and nodal MZBCL [7]. The ‘MALT concept’ for lymphoid infiltrates in the gastric or intestinal mucosa described by Issacson and Wright in 1983 provides the basis for the definition of extranodal ‘MALT-type’ MZBCL, which differs from MZBCL of nodal or splenic origin [7]. Histologically, extranodal MALT-type MZBCL has a characteristic morphology with lymphoepithelial lesions induced by invading lymphocytes [7] and, usually, poorly defined follicular appearing areas (diffuse) with heterogeneous cellular infiltrates comprising centrocyte-like cells, monocytoid B cells, small lymphocytes, and plasma cells [8].

The immunophenotype of the neoplastic lymphocytes was determined by immunohistochemistry. Proliferating B cells can be identified with immunohistochemistry and are expected to be positive for CD20 and CD79a but should be negative for CD10, CD3, and CD5. Approximately 70% of canine lymphoma cases are B-cell lymphomas, and typically, B-cell type is less aggressive than T-cell type lymphoma. Diffuse large B-cell lymphoma is the most common type of canine lymphoma, and follicular lymphoma (centroblastic [CB]/centrocytic [CC]) is also common but is more likely to be disseminated and has a rare primary occurrence [6].

Extranodal marginal zone B-cell lymphoma tends to occur in patients with a history of autoimmune disease and chronic inflammatory disorders [2]. This chronic inflammation induces accumulation of lymphoid tissue. Primary gastric extranodal MALT-type MZBCL is usually preceded by pre-existing *Helicobacter pylori* infection in human cases, and low-grade MALT-type MZBCL in the stomach is likely to respond to eradication of the *H. pylori* infection [3].

When gastric MALT-type lymphoma is localised, the patient may be treated with surgery or radiotherapy and antimicrobial therapy for pre-existing chronic inflammation; however, if the disease is disseminated and at a more advanced stage, single- or multi-agent chemotherapy may be required [2]. There is currently no standardised treatment for non-gastrointestinal extranodal marginal zone B-cell lymphoma. However, both gastric and non-gastric MZBCL are similar in their development and pathologic manifestations, so administering a similar treatment to non-gastrointestinal extranodal MZBCL may be effective.

In the present study, we described a rare uterine tumour identified as primary extranodal MZBCL in a dog, and, to our knowledge, this unusual presentation has not been reported previously in the veterinary literature. Although future studies are required for further pathologic analysis, this report may help improve the diagnosis and clinical approach for rare uterine tumours of dogs.

## Endnotes

^a^Vector Laboratories, Inc., Burlingame, CA, USA

^b^Abcam, plc., Cambridge, UK

^c^Santa Cruz Biotechnology, Inc., Dallas, Texas, USA

^d^Zymed laboratories, Inc., South San Francisco, CA, USA

## Competing interests

The authors declare that they have no competing interests.

## Authors’ contributions

JS evaluated the tissue sample by HE stain and immunohistochemistry and drafted the manuscript. HJ participated in tissue preparation and prepared a paraffin-embedded section. S performed the ovariohysterectomy procedure and reported clinical findings. SH supported the histopathological diagnosis and supervised the manuscript. All authors read and approved the final manuscript.
